# Unmet Needs in Managing Myocardial Infarction in Patients With Malignancy

**DOI:** 10.3389/fcvm.2019.00057

**Published:** 2019-05-17

**Authors:** Taku Inohara, Ayaka Endo, Chiara Melloni

**Affiliations:** ^1^Duke Clinical Research Institute, Duke University Medical Center, Durham, NC, United States; ^2^Department of Cardiology, Keio University School of Medicine, Tokyo, Japan; ^3^Department of Cardiology, Saiseikai Central Hospital, Tokyo, Japan

**Keywords:** myocardial infarction, cancer, arterial thrombosis, chemotherapy, invasive strategy

## Abstract

Patients with cancer face a high short-term risk of arterial thromboembolism. One of the most fatal manifestations of arterial thromboembolism is myocardial infarction (MI), and patients with cancer face a 3-fold greater risk of MI than patients without cancer. The individual risk for arterial thrombotic events in patients with cancer is determined by the complex interaction of baseline cardiovascular risk factors, cancer type and stage, chemotherapeutic regimen, and other general contributing factors for thrombosis. Managing MI in patients with cancer is a clinical challenge, particularly due to cancer's unique pathophysiology, which makes it difficult to balance thrombotic and bleeding risks in this specific patient population. When patients with cancer present with MI, a limited proportion are treated with guideline-recommended therapy, such as antiplatelet therapy or invasive revascularization. Despite the limited evidence, existing reports consistently suggest similar clinical benefits of guideline-recommended therapy when administered to patients with cancer presenting with MI. In this review, we briefly summarize the available evidence, clinical challenges, and future perspectives on simultaneous management of MI and cancer, with a focus on invasive strategy.

## Introduction

Advances in cancer treatments have significantly contributed to a decline in cancer-specific mortality rates; as a result, cardiovascular disease has become the leading cause of death among cancer patients (1). The incidence of myocardial infarction (MI), in particular, is higher in patients with active cancer compared to those without cancer (2). The increased risk of thrombotic events in cancer patients is partially attributable to the pro-coagulant state in this population, and partially due to the adverse effects of some chemotherapeutic agents (2). Data from a nationwide Swedish report indicated that the overall incidence of coronary heart disease was 152 per 100,000 person year in patients with cancer compared with 143 per 100,000 person year in patients without cancer, with the risk of heart disease being more prominent during the first 6 months post-cancer diagnosis than in the periods following this 6-month time window (3). Navi et al. reported similar findings from the Surveillance, Epidemiology, and End Results (SEER)–Medicare database; patients with cancer have a 3-fold greater risk of MI when compared with patients without cancer (6-month cumulative incidence of 2.0 vs. 0.7%, respectively; hazard ratio, HR, 2.9 [95% confidence interval, CI, 2.8–3.1]) (4). Although there is increasing recognition that cancer and heart disease coexist, there are limited data on how to optimally manage these high-risk patients with both comorbidities. In this mini review, we provide an overview of the pathophysiologic mechanism, presentation, and management of MI in patients with cancer, with a particular focus on invasive strategy.

### Possible Mechanisms Causing Increased MI Risk in Patients With Cancer

The individual risk for arterial thrombotic events in cancer patients is determined by the complex interaction of multiple elements including baseline cardiovascular risk factors, cancer type and stage, chemotherapeutic regimen, and other general contributing factors for thrombosis (2). Conventional cardiovascular risk factors such as age, hypertension, dyslipidemia, smoking, and diabetes are often present in cancer patients. Lupus anticoagulant and hyperhomocysteinemia are other known contributing risk factors of arterial thrombosis (5). Additionally, the risk of arterial thrombosis substantially varies among different types of cancer. Navi et al. have shown that patients with lung, gastric, or pancreatic cancers had the highest rates of arterial thrombotic events, including MI and stroke (4). Among hematologic malignancies, patients with multiple myeloma have an increased risk of arterial thrombosis (6).

**Figure 1:**
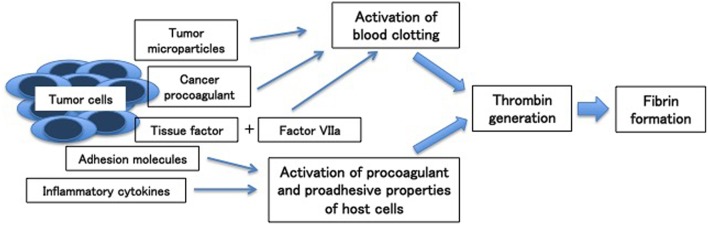
Mechanisms of cancer-associated thrombosis [Adapted from (7)].

Aside from clinical factors, biological factors produced by tumor cells also contribute to activate hemostatic system in cancer patients. Cancer cells can activate the hemostatic system through the expression of pro-coagulant factors including tissue factor and cancer pro-coagulant, release of inflammatory cytokines (i.e., TNF-α, IL-1β) and microparticles, and adhesion to host vascular cells (Figure 1) (7–9).

Tissue factor, constitutively expressed on the malignant cell surface of a variety of cancers, plays an important role in thrombin by forming a complex with factor VIIa, triggering blood coagulation by activating factor IX and factor X. Unlike tissue factor, cancer procoagulant, another procoagulant factor, directly activates factor X independently of factor VII.

Microparticles, small plasma membrane vesicles, are also released by tumor cells and associated with hypercoagulable state of cancer. Their mechanism is related to intravascular thrombin generation by exposure of phosphatidylserine and procoagulant proteins such as tissue factor. In addition, tumor cells release inflammatory cytokines, which can stimulate the prothrombotic features of vascular cells.

Several chemotherapy agents are known to increase the risk of myocardial ischemia through different mechanisms, including direct vasospastic effect, endothelial injury, acute arterial thrombosis, and the long-term effect on lipid metabolism, resulting in plaque destabilization. (Table 1) (5, 10, 11) For example, some cancer therapies cause endothelial damage resulting in arterial thrombosis. There are two main groups of chemotherapeutics that may have a detrimental effect on the function of endothelial cells: VEGF inhibitors and Bcr-Abl tyrosine kinase inhibitors. VEGF is an essential factor for endothelial cells to grow and survive. Inhibiting the VEGF pathway causes devastating injury to endothelial cells, resulting in progression of atherosclerosis, provocation of ischemia, and arterial thrombosis (2). The Bcr-Abl signaling pathway synergistically works with the VEGF pathway and plays a vital role in endothelial cell survival; therefore, chemotherapeutics for hematologic malignancies (e.g., chronic myeloid leukemia) that inhibit Bcr-Abl inhibitors result in an increased risk of arterial thrombosis (2).

**Table 1:** Pathophysiological mechanisms of myocardial ischemia related to cancer treatment.

**Agent**	**Pathophysiological mechanism**
Fluoropyrimidines (5-FU, capecitabine, gemcitabine)	Endothelial injury Vasospasm
Anti-microtubule agents (paclitaxel, docetaxel)	Vasospasm
Platinum compounds (cisplatin)	Procoagulant status Arterial thrombosis
Antitumor antibody (bleomycin)	Endothelial injury
Vinca alkaloids (vincristine)	Endothelial apoptosis
VEGF inhibitors (bevacizumab, sorafenib, sunitinib)	Procoagulant status Arterial thrombosis Endothelial injury
Radiotherapy	Endothelial injury Plaque rupture Thrombosis

Capecitabine- and 5-fluorouracil (5-FU)-associated cardiotoxicity is often overlooked, but both drugs are known to increase the risk of MI, especially when administered with a continuous 24-h 5-FU + leucovorin infusion for 5 days (12). Reports have also indicated that cisplatin is linked to an increased risk of MI. Moore et al. reported that 20 out of 932 patients who were treated with cisplatin-based chemotherapy experienced arterial thrombosis, including 2 MIs (13). Some molecular target drugs have also been implicated in the occurrence of MI. In patients with metastatic colorectal, breast, or non-small cell lung carcinoma, the combination therapy of bevacizumab, humanized monoclonal antibody against VEGF, and chemotherapy has been associated with an increased risk of arterial thrombosis compared with chemotherapy alone (5.5 vs. 3.1 events per 100 person-years) (14). A meta-analysis demonstrated that arterial thromboembolic events occurred more frequently in patients treated with sunitinib and sorafenib (which are VEGF receptor tyrosine kinase inhibitors) than in control patients, with an overall incidence of 1.7% for sorafenib and 1.4% for sunitinib (15). Radiotherapy, particularly applied to the supradiaphragmatic area, is also known to be associated with an increased risk of ischemic heart disease, mostly due to ostial lesions (16).

### Clinical Manifestation

Cancer patients may experience different presentations of an acute MI. A University of Texas MD Anderson Cancer Center report highlighted how symptoms can be atypical in cancer patients, with dyspnea (rather than chest pain) being the most common presentation (17). Furthermore, of 456 cancer patients diagnosed with MI, 85% of them had non-ST-segment elevation MI (NSTEMI) and 15% had ST-segment elevation MI (STEMI) (17). As a result of these findings, patients with cancer who present with atypical symptoms (such as dyspnea) should be screened for acute MI. The initial cardiac workup for acute MI, which is based on repeated electrocardiograms and cardiac biomarkers, should be the same for patients with and without cancer. Munoz et al. found that >10% of cancer patients with acute coronary syndrome (ACS) presentation were ultimately diagnosed with Takotsubo cardiomyopathy, which is a non-ischemic transient cardiac syndrome that does not require antithrombotic therapy (18). Distinguishing between ACS and other cardiac syndromes like Takotsubo cardiomyopathy is important, since identifying the appropriate cardiac diagnosis may allow unnecessary antithrombotic medications (such as unfractionated heparin or P2Y_12_ inhibitors) to be discontinued in order to avoid unnecessary bleeding.

### How to Manage MI in Cancer Patients

#### Prevention

Ischemic workup should be done in high-risk patients to detect pre-existing coronary artery disease, which is known to be a risk factor for chemotherapy-induced MI, before administering cancer drugs known to cause cardiac ischemia (Table 1) (19).

#### Medical Therapy

There are limited data that sufficiently address the management of cardiac ischemic disease in patients with cancer. Especially in an acute phase, there is scant data regarding optimal management for cancer patients presenting with MI. Further studies are warranted to clarify optimal antithrombotic regimen, such as unfractionated heparin vs. low molecular heparin vs. bivalirudin. Aspirin and beta-blockers are the primary drugs used to treat patients with MI in acute and chronic settings, but it is unknown if the safety and efficacy of aspirin and beta-blockers is equally preserved in cancer patients. Yusuf et al. retrospectively analyzed 456 cancer patients with MI, including 70 who presented with STEMI (17); of these patients, only 211 (46.3%) received aspirin. One-year survival was higher in patients treated with aspirin (34%) than in those without (18%). After adjustment for demographic baseline differences, aspirin use was significantly associated with improved survival at 1-year. Similarly, less than half (48.5%) of patients were treated with a beta-blocker, and 1-year survival was higher in those who received a beta-blocker (36.0%) compared with those who did not (16.0%). The survival benefit persisted even after multiple adjustments. Yusuf et al. also evaluated the efficacy of statin and angiotensin-converting enzyme inhibitors, but they were not associated with improved survival at 1 year. Despite a relatively small sample size and unmeasured confounders, Yusuf et al.'s study provides persuasive evidence in support of using aspirin and a beta-blocker when cancer patients suffer from MI. The National Registry of Acute Myocardial Infarction in Switzerland (AMIS Plus) reported consistently reduced standard of care treatment for MI in patients with cancer vs. those without (20); consequently, further efforts are required to facilitate rigorous implementation of these cardioprotective medications to improve patient outcomes.

#### Invasive Management

Cancer patients with MI are less likely to be treated with catheter-based revascularization (i.e., PCI), even for STEMI. The MD Anderson Cancer Center found that among 456 cancer patients with MI, only 11 (2.8%) underwent PCI. Of those presenting with STEMI, only 5.7% underwent PCI (17). Pothineni et al. analyzed the United States National Inpatient Sample and found that the utilization of PCI in STEMI patients with cancer varied according to the type of cancer, ranging from 17.3% in colon cancer to 30.8% in breast cancer (21). In the National Registry of Acute Myocardial Infarction in Switzerland (AMIS Plus), which is a large Swiss registry of patients with ACS, patients with a history of cancer were less likely to undergo PCI than those without (67.8 vs. 73.4%; adjusted odds ratio [OR] 0.76, 95% CI 0.67–0.88) (20). Notably, patients treated with PCI were less likely to die than those who did not receive revascularization (17, 21). There are a couple potential reasons for why a minority of patients with cancer received revascularization therapy: (1) a poor prognosis due to the cancer itself; and/or (2) concern for bleeding complications with prolonged dual antiplatelet therapy (DAPT) resulting from stent implantation. Due to advances in stent technology, the recommended duration of DAPT after stent implantation has been getting shorter, which may allow the PCI indication for cancer patients with MI to be expanded (22).

To date, there are several moderate-scale observational studies comparing clinical outcomes between patients with and without a history of cancer undergoing PCI (23–26). The CREDO-Kyoto Registry Cohort-2 (*N* = 12,180; history of cancer, 9.1%; ACS, 27.2%) found that the cumulative 5-year incidences of cardiovascular death were significantly higher in a group of patients with cancer vs. one without (12.4 vs. 7.5%, *p* < 0.001) (23). Even after adjustment, the excess risk of cardiovascular death in the cancer group relative to the non-cancer group remained significant. Findings were similar for other cardiovascular-related outcomes—the adjusted risks for all-cause death, non-cardiac death, heart failure readmission, and major bleeding were higher in cancer patients than in non-cancer patients, while the risks for MI and stroke were not different between groups. Subgroup analysis in ACS patients (*N* = 3,309, 27.2%) demonstrated consistent findings with those in the main analysis.

The Bleeding Complications in a Multicenter Registry of Patients Discharged after an Acute Coronary Syndrome (BleeMACS) project (*N* = 15,401; history of cancer, 6.4%), which was an international multicenter observational registry with 15 participating hospitals. BleeMACS compared the clinical outcomes of patients with and without cancer, who were also diagnosed with ACS and treated with PCI (25). After 1 year of follow-up, adverse cardiovascular events (i.e., a composite of all-cause death, MI, and bleeding events) and bleeding were significantly higher in cancer patients than non-cancer patients (adverse cardiovascular events: 15.2 vs. 5.3%; bleeding: 6.5 vs. 3.0%). After adjustment, the increased risks of adverse cardiovascular and bleeding events in cancer patients remained significant. An analysis from the Duke database (*N* = 15,008; history of cancer, 3.3%; ACS, 72.0%) found that after a 14-year follow-up, cardiovascular mortality was not different between groups (31.4 vs. 27.7%, *p* = 0.31), but the rate of all-cause death was significantly higher in patients with a history of cancer than in those without (79.7 vs. 49.3%, *p* < 0.01) (24). These varying results are likely due to differences in the definitions of cancer and outcomes, as well as differences in study population and follow-up duration.

When PCI is considered as a treatment option for cancer patients presenting with MI, higher risk of stent thrombosis should be taken into account. Several registries have demonstrated an underlying hypercoagulable state that predisposes cancer patients to a higher risk of stent thrombosis. The Dutch Stent Thrombosis Registry found that active cancer was associated with stent thrombosis (27). Among 437 patients diagnosed with definite stent thrombosis, 46 patients (10.5%) had active cancer. Similarly, the Coronary Revascularization Demonstrating Outcome Study in Kyoto (CREDO-Kyoto) Percutaneous Coronary Intervention (PCI)/Coronary Artery Bypass Grafting (CABG) Registry Cohort-2 reported that patients with active cancer who were undergoing PCI trended toward higher adjusted risk for definite or probable stent thrombosis as compared with patients without cancer, although this finding did not reach statistical significance (23). A retrospective chart review of patients treated with a bare metal stent at a single center in Germany also reported a higher rate of in-stent thrombosis in patients with cancer compared with those without cancer (5.6 vs. 0.8%) (28).

#### Intervention: Choice of Devices

Stent implantation undoubtedly remains the gold standard of PCI. Bare metal stents (BMS) were initially favored for patients with a high bleeding risk who were not able to tolerate prolonged DAPT; however, due to the development of drug-eluting stents (DES), prolonged duration of DAPT after DES placement is no longer necessary. The 2017 European Society of Cardiology Focused Update on Dual Antiplatelet Therapy in Coronary Artery Disease recommends 6 months of DAPT after stent implantation post-ACS event in patients with a high bleeding risk, regardless of stent type (22). In the 2016 American College of Cardiology (ACC)/American Heart Association (AHA) Focused Update on Duration of Dual Antiplatelet Therapy in Patients with Coronary Artery Disease, at least 1 year of DAPT is recommended for ACS patients treated with PCI, regardless of stent type (Class I), and P2Y_12_ therapy discontinuation after 6 months is considered a reasonable option for patients with high bleeding risk (Class IIb) (29). The other main concern surrounding DES deployment was a higher risk of stent thrombosis, but this concern has already been resolved by the development of second-generation DES (30). Given these improvements in DES, there may be no reason for physicians to recommend BMS over DES, even for those patients whose cardiac treatment is complicated by cancer.

Drug-coated balloon (DCB) is also an emerging technology in the field of PCI. Recently, the Basel Kosten Effektivitäts Trial–Drug-Coated Balloons vs. Drug-Eluting Stents in Small Vessel Interventions (BASKET-SMALL2) trial demonstrated the non-inferiority of DCB compared to DES regarding major adverse cardiovascular events (composite of cardiac death, non-fatal MI, and target-vessel revascularization) up to 12 months in PCIs for *de novo* lesions (<3 mm in diameter) in coronary vessels (31). This emerging technology could provide a novel management approach to MI, particularly for cancer patients in whom prolonged antiplatelet therapy often poses a major clinical dilemma. Nevertheless, current manufacturers still recommend 3 months of DAPT following DCB treatment; therefore, the clinical benefit of DCB over metallic stent when treating cancer patients presenting with MI is negated.

#### Intervention: Access Site

Another important key to reducing bleeding complications is appropriate access site selection. Given the high bleeding risk in patients with cancer, appropriate access site selection is particularly critical. In terms of reducing bleeding complications, radial access is generally considered favorable to femoral. There is no favored access site recommendation in the current 2013 American College of Cardiology Foundation (ACCF)/AHA Guideline for the Management of ST-Elevation Myocardial Infarction (32, 33), but in the 2017 ESC Guidelines for the Management of Acute Myocardial Infarction in Patients Presenting with ST-Segment Elevation, radial access is recommended for PCI in patients with ACS, including Class I STEMI (34, 35). Femoral access needs to be considered for PCI in hemodialysis patients or in patients in whom radial access is difficult to obtain. Every effort should be taken to avoid access site complications, such as the use of smaller sheath sizes, a lower dose of intra-arterial or intravenous unfractionated heparin, or a femoral angiogram after PCI (36). Due to the high-risk profile of bleeding complications, the Society for Cardiovascular Angiography and Interventions (SCAI) Expert Consensus Statement recommends a transradial approach for cancer patients who are excellent candidates for both radial and femoral access (36).

#### Patient With Thrombocytopenia

Cancer patients frequently develop thrombocytopenia after chemotherapy, with an incidence ranging from 10 to 25% (37). The standard approaches to treating an MI, such as antiplatelet, anticoagulant, and thrombolytic therapies exacerbate bleeding risk and, consequently, are typically withheld from patients with thrombocytopenia. Nonetheless, accumulating evidence may support the implementation of these standard approaches—even for this specific population. In hopes of addressing the efficacy and safety of antiplatelet therapy in MI patients with thrombocytopenia, investigators from the MD Anderson Cancer Center reported a case series (38) that demonstrated how, in patients with thrombocytopenia, the risk of bleeding varies and may depend on the underlying cause, instead of absolute antiplatelet counts. The investigators also showed that 7-day survival was higher in patients who received aspirin vs. those who did not (90 vs. 6%), although treatment selection bias should be taken into account (39). Iliescu et al. reviewed 30 cases with chronic thrombocytopenia (defined as absolute platelet count <100,000/mm^3^) who underwent coronary stenting and found that all procedures were completed without major bleeding complications and platelet transfusion (40). The SCAI Expert Consensus Statement recommends not to transfuse platelets prophylactically in cancer patients undergoing cardiac catheterization with thrombocytopenia, unless platelet counts are <20,000/ml and the oncology/hematology team recommends transfusion (36). SCAI also encourages reduced platelet count thresholds for cardiovascular therapies, recommending aspirin initiation in patients with platelet counts >10,000/ml and DAPT initiation (with aspirin and clopidogrel) if platelet counts are >30,000/ml. Due to a lack of evidence, prasugrel, ticagrelor, and glycoprotein IIb/IIIa inhibitors should not be used in patients with platelet counts <50,000/ml (36).

## Conclusion

In cancer patients, endothelial dysfunction that is caused by the cancer cell(s) itself, as well as chemotherapeutics, promotes platelet aggregation, which results in an increased risk of arterial thrombosis (including MI). Optimal management of MI among patients with cancer remains a clinical challenge. Available evidence is only derived from small- or moderate-sized observational data; there are currently no randomized clinical trials investigating the optimal management of cancer patients at high risk for thrombosis. The studies that do exist contain inconsistent definitions of cancer, making it difficult to compare them to each other, and challenging to combine datasets. Looking to the future, prospective studies that involve cardiologists and oncologists who agree to universal definitions of MI and cancer will enhance our understanding of the best way to treat this high-risk population. Device improvement may enable the wide application of invasive management to this specific patient population, but further evidence is needed to optimally treat patients with cancer and MI.

## Author Contributions

TI and CM contributed the study concept and design. TI contributed the manuscript drafting. AE and CM contributed the critical revision of the manuscript for important intellectual content, as well as the study supervision.

### Conflict of Interest Statement

TI discloses the following relationships—Research Grant: JSPS Overseas Research fellowship and Boston Scientific. CM's disclosure can be viewed in the Supplementary Data Sheet 1. The remaining author declares that the research was conducted in the absence of any commercial or financial relationships that could be construed as a potential conflict of interest.

## Supplementary Material

Data Sheet 1 CM's disclosure.
