# Supplementary material for: Unmet Needs in Managing Myocardial Infarction in Patients With Malignancy
Source: Front Cardiovasc Med. 2019 May 17;6:57. doi: 10.3389/fcvm.2019.00057 (PMC6533845; doi:10.3389/fcvm.2019.00057)
Supplement: Data Sheet 1 — CM's disclosure. [file Data_Sheet_1.pdf]

DCRI COMMERCIAL RELATIONSHIPS TRACKING FORM

Duke COI Reporting is based on the previous year and anticipated relationships for the coming year. Please report current and anticipated information for the calendar year (January through December) 2017. For audit purposes, please keep conflict of interest/financial records for three years after the conclusion of the award.

How to Use This Form:

1) Save the document on your drive.
2) Enter the Year (e.g. 2016), your Name, Title and Address in the appropriate fields.
3) Make sure you save your version on your drive.
4) Then Click on “File”, click “Protect Document”, in the drop-down menu click “Restrict Editing”. In the column to the right at the bottom click “unprotect”.
5) Click on the appropriate checkboxes.
6) Save your updated version on your drive
7) Send an email to DCRI Communications (DCRIExternalWebsite@dm.duke.edu) with your COI attached for posting on our DCRI website.

Editing the Document (Necessary only when you have to add companies not on the form):

1) Scroll down to the last page to the blank rows and enter the company name(s) on the company field(s).
2) Enter new information.
3) Click on the appropriate checkboxes on your new listing and save.
4) Make sure you save your version on your drive.

Column Explanations:

(1) Funding is provided for my effort in research grants or contracts for multi-center projects in which I serve as either the Chairman, Co-Chairman, Executive or Steering Committee member or I am involved as a DCRI faculty leader for specific aspects in a project. A check in this column denotes that I am involved in such a research contract. Payments for the work performed for these contracts are made to the University. A portion of my salary as a faculty member, representative of % effort on the project, is supported by these funds.

(2) A research grant from this company supports the expenses associated with projects in the DUHS that are under my direction.

(3) I provide educational activities or deliver occasional lectures that generate revenue from this company. All revenues from these activities are paid to the University. None of it is taken for personal income. The three columns represent annual payments of more than $5,000, between $5,000 and $25,000, and in excess of $25,000 per year from this company.

(4) I receive personal income outside of my University salary for consulting or other services (Including CME) from this company. The three columns represent annual payments of more than $5,000, between $5,000 and $25,000, and in excess of $25,000 per year from this company.

(5) I receive personal income outside of my University salary for consulting or other non-CME services from this company. The three columns represent annual payments of more than $5,000, between $5,000 and $25,000, and in excess of $25,000 per year from this company.

(6) I personally receive royalties in excess of $5,000 per year from this company.

(7) I, or an immediate member of my family, have equity (with shareholder voting privileges) in this company of either > $5,000 or > 1%.

***If you have no conflict to report please check the box at the end of the list.

DCRI COMMERCIAL RELATIONSHIPS TRACKING FORM

Year: 2017

Name: Chiara Melloni, MD FACC

Title: Assistant Professor of Medicine, Cardiology

Address: Duke Clinical Research Institute, 2400 Pratt Street, Durham, NC 27705

Please complete the applicable rows in the following table. Monetary amounts are on an annual basis.

Company:	(1) A Research Grant or Contract from this Company Partially Supports My University Salary	(2) A Research Grant or Contract from this Company Supports My Research Projects	(3) Educational Activities or Lectures for this Company Generates *Revenue for Duke*			(4) Consulting or Other Services (Including CME) for this Company Generates *Personal Income*			(5) Consulting or Other non-CME Services for this Company Generates *Personal Income*			(6) I Receive Significant Personal Royalties from this Company	(7) I Have Equity in this Company	
			<$5K	$5K–$25K	>$25K	<$5K	$5K–$25K	>$25K	<$5K	$5K–$25K	>$25K		<$5K	>$5K/1%
Abbott Laboratories	☐	☐	☐	☐	☐	☐	☐	☐	☐	☐	☐	☐	☐	☐
Abiomed	☐	☐	☐	☐	☐	☐	☐	☐	☐	☐	☐	☐	☐	☐
Acerta Pharma	☐	☐	☐	☐	☐	☐	☐	☐	☐	☐	☐	☐	☐	☐
Achillion Pharmaceuticals	☐	☐	☐	☐	☐	☐	☐	☐	☐	☐	☐	☐	☐	☐
Acorda Therapeutics, Inc	☐	☐	☐	☐	☐	☐	☐	☐	☐	☐	☐	☐	☐	☐
Aires Pharmaceuticals	☐	☐	☐	☐	☐	☐	☐	☐	☐	☐	☐	☐	☐	☐
Akili Interactive Labs	☐	☐	☐	☐	☐	☐	☐	☐	☐	☐	☐	☐	☐	☐
Alcobra	☐	☐	☐	☐	☐	☐	☐	☐	☐	☐	☐	☐	☐	☐
Amgen, Inc.	☒	☐	☐	☐	☐	☐	☐	☐	☐	☐	☐	☐	☐	☐
Amylin Pharmaceuticals, Inc.	☐	☐	☐	☐	☐	☐	☐	☐	☐	☐	☐	☐	☐	☐
ARCA Biopharma	☐	☐	☐	☐	☐	☐	☐	☐	☐	☐	☐	☐	☐	☐
Ardea Biosciences, Inc.	☐	☐	☐	☐	☐	☐	☐	☐	☐	☐	☐	☐	☐	☐
Armetheon Inc	☐	☐	☐	☐	☐	☐	☐	☐	☐	☐	☐	☐	☐	☐

AstraZeneca	☒	☐	☐	☐	☐	☐	☐	☐	☐	☐	☐	☐	☐	☐
Atentiv Inc	☐	☐	☐	☐	☐	☐	☐	☐	☐	☐	☐	☐	☐	☐
Baseline Study LLC	☐	☐	☐	☐	☐	☐	☐	☐	☐	☐	☐	☐	☐	☐
Baxalta	☐	☐	☐	☐	☐	☐	☐	☐	☐	☐	☐	☐	☐	☐
Bayer Corporation US (Bayer AG/Bayer in Japan – subsidiaries)	☐	☐	☐	☐	☐	☐	☐	☐	☐	☐	☐	☐	☐	☐
Beckman Research Institiute	☐	☐	☐	☐	☐	☐	☐	☐	☐	☐	☐	☐	☐	☐
Bellerophon Therapeutics	☐	☐	☐	☐	☐	☐	☐	☐	☐	☐	☐	☐	☐	☐
Biogen	☐	☐	☐	☐	☐	☐	☐	☐	☐	☐	☐	☐	☐	☐
Biogen Idec In	☐	☐	☐	☐	☐	☐	☐	☐	☐	☐	☐	☐	☐	☐
Biomarin	☐	☐	☐	☐	☐	☐	☐	☐	☐	☐	☐	☐	☐	☐
Biosensors International Group Ltd	☐	☐	☐	☐	☐	☐	☐	☐	☐	☐	☐	☐	☐	☐
BioStealth Inc	☐	☐	☐	☐	☐	☐	☐	☐	☐	☐	☐	☐	☐	☐
Boehringer Ingelheim	☐	☐	☐	☐	☐	☐	☐	☐	☐	☐	☐	☐	☐	☐
Boston Scientific Corporation	☐	☐	☐	☐	☐	☐	☐	☐	☐	☐	☐	☐	☐	☐
Bristol Myers Squibb	☒	☐	☐	☐	☐	☐	☐	☐	☐	☐	☐	☐	☐	☐
Burroughs Welcome Fund	☐	☐	☐	☐	☐	☐	☐	☐	☐	☐	☐	☐	☐	☐
Cardio3 Bioscience	☐	☐	☐	☐	☐	☐	☐	☐	☐	☐	☐	☐	☐	☐
CardioDx, Inc.	☐	☐	☐	☐	☐	☐	☐	☐	☐	☐	☐	☐	☐	☐
Cardiovascular Systems, Inc.	☐	☐	☐	☐	☐	☐	☐	☐	☐	☐	☐	☐	☐	☐
Cempra Pharmaceuticals, Inc.	☐	☐	☐	☐	☐	☐	☐	☐	☐	☐	☐	☐	☐	☐
Cerenova, LLC	☐	☐	☐	☐	☐	☐	☐	☐	☐	☐	☐	☐	☐	☐
CPC Clinical Research	☐	☐	☐	☐	☐	☐	☐	☐	☐	☐	☐	☐	☐	☐

CSL Group	☐	☐	☐	☐	☐	☐	☐	☐	☐	☐	☐	☐	☐	☐
CSL Limited	☐	☐	☐	☐	☐	☐	☐	☐	☐	☐	☐	☐	☐	☐
Cubist Pharmaceuticals	☐	☐	☐	☐	☐	☐	☐	☐	☐	☐	☐	☐	☐	☐
Daiichi Sankyo Co. Limited	☐	☐	☐	☐	☐	☐	☐	☐	☐	☐	☐	☐	☐	☐
Deloitte Consulting LLP	☐	☐	☐	☐	☐	☐	☐	☐	☐	☐	☐	☐	☐	☐
Dr. Reddy's Laboratories LTD	☐	☐	☐	☐	☐	☐	☐	☐	☐	☐	☐	☐	☐	☐
Duke Clinical Research Institute	☐	☐	☐	☐	☐	☐	☐	☐	☐	☐	☐	☐	☐	☐
Duke Neurology Imaging Lab	☐	☐	☐	☐	☐	☐	☐	☐	☐	☐	☐	☐	☐	☐
Durata Therapeutics	☐	☐	☐	☐	☐	☐	☐	☐	☐	☐	☐	☐	☐	☐
Edwards Lifesciences	☐	☐	☐	☐	☐	☐	☐	☐	☐	☐	☐	☐	☐	☐
Elesvier	☐	☐	☐	☐	☐	☐	☐	☐	☐	☐	☐	☐	☐	☐
Eli Lilly & Company	☐	☐	☐	☐	☐	☐	☐	☐	☐	☐	☐	☐	☐	☐
Evalve, Inc	☐	☐	☐	☐	☐	☐	☐	☐	☐	☐	☐	☐	☐	☐
Exelixis, Inc.	☐	☐	☐	☐	☐	☐	☐	☐	☐	☐	☐	☐	☐	☐
F Hoffmann-La Roche Ltd	☐	☐	☐	☐	☐	☐	☐	☐	☐	☐	☐	☐	☐	☐
Ferring Pharmaceuticals	☒	☐	☐	☐	☐	☐	☐	☐	☐	☐	☐	☐	☐	☐
Genentech	☐	☐	☐	☐	☐	☐	☐	☐	☐	☐	☐	☐	☐	☐
General Electric Company	☐	☐	☐	☐	☐	☐	☐	☐	☐	☐	☐	☐	☐	☐
General Electric Healthcare	☐	☐	☐	☐	☐	☐	☐	☐	☐	☐	☐	☐	☐	☐
Genetic Alliance	☐	☐	☐	☐	☐	☐	☐	☐	☐	☐	☐	☐	☐	☐
Genzyme Corporation	☐	☐	☐	☐	☐	☐	☐	☐	☐	☐	☐	☐	☐	☐
Gilead Sciences, Inc.	☐	☐	☐	☐	☐	☐	☐	☐	☐	☐	☐	☐	☐	☐
Glaxo SmithKline (GSK)	☒	☐	☐	☐	☐	☐	☐	☐	☐	☐	☐	☐	☐	☐

Grifols	☐	☐	☐	☐	☐	☐	☐	☐	☐	☐	☐	☐	☐	☐
Harvard Pilgrim Health Care	☐	☐	☐	☐	☐	☐	☐	☐	☐	☐	☐	☐	☐	☐
HeartFlow	☐	☐	☐	☐	☐	☐	☐	☐	☐	☐	☐	☐	☐	☐
Hologic, Inc.	☐	☐	☐	☐	☐	☐	☐	☐	☐	☐	☐	☐	☐	☐
Humacyte, Inc	☐	☐	☐	☐	☐	☐	☐	☐	☐	☐	☐	☐	☐	☐
Immuron Ltd	☐	☐	☐	☐	☐	☐	☐	☐	☐	☐	☐	☐	☐	☐
Janssen Pharmaceutica Products, L.P.	☐	☐	☐	☐	☐	☐	☐	☐	☐	☐	☐	☐	☐	☐
Janssen Research and Development, LLC														
Janssen Scientific Affairs, LLB	☐	☐	☐	☐	☐	☐	☐	☐	☐	☐	☐	☐	☐	☐
Johnson & Johnson	☐	☐	☐	☐	☐	☐	☐	☐	☐	☐	☐	☐	☐	☐
Johnson & Johnson Pharmaceutical Research & Development	☐	☐	☐	☐	☐	☐	☐	☐	☐	☐	☐	☐	☐	☐
LadeRX	☐	☐	☐	☐	☐	☐	☐	☐	☐	☐	☐	☐	☐	☐
Luitpold Pharmaceuticals, Inc.	☒	☐	☐	☐	☐	☐	☐	☐	☐	☐	☐	☐	☐	☐
MAQUET Cardiovascular LLC (formerly Boston Scientific Cardiac & Vascular)	☐	☐	☐	☐	☐	☐	☐	☐	☐	☐	☐	☐	☐	☐
Marinus Pharmaceuticals Inc	☐	☐	☐	☐	☐	☐	☐	☐	☐	☐	☐	☐	☐	☐
Medical Education Solutions Group	☐	☐	☐	☐	☐	☐	☐	☐	☐	☐	☐	☐	☐	☐
MedImmune Inc	☐	☐	☐	☐	☐	☐	☐	☐	☐	☐	☐	☐	☐	☐
Medscape, LLC	☐	☐	☐	☐	☐	☐	☐	☐	☐	☐	☐	☐	☐	☐
Medtronic PLC	☐	☐	☐	☐	☐	☐	☐	☐	☐	☐	☐	☐	☐	☐

Medtronic Vascular, Inc. (formerly Medtronic AVE)	☐	☐	☐	☐	☐	☐	☐	☐	☐	☐	☐	☐	☐	☐
Merck & Co., Inc	☒	☐	☐	☐	☐	☐	☐	☐	☐	☐	☐	☐	☐	☐
Merck Sharp & Dohme I.A. Corporation	☐	☐	☐	☐	☐	☐	☐	☐	☐	☐	☐	☐	☐	☐
MyoKardia Inc	☐	☐	☐	☐	☐	☐	☐	☐	☐	☐	☐	☐	☐	☐
Novartis Consumer Health Inc	☐	☐	☐	☐	☐	☐	☐	☐	☐	☐	☐	☐	☐	☐
Novartis Pharmaceutical Company	☐	☐	☐	☐	☐	☐	☐	☐	☐	☐	☐	☐	☐	☐
Novavax Inc	☐	☐	☐	☐	☐	☐	☐	☐	☐	☐	☐	☐	☐	☐
Ophthotech Corporation	☐	☐	☐	☐	☐	☐	☐	☐	☐	☐	☐	☐	☐	☐
OrbusNeich	☐	☐	☐	☐	☐	☐	☐	☐	☐	☐	☐	☐	☐	☐
Otsuka America Pharmaceutical	☐	☐	☐	☐	☐	☐	☐	☐	☐	☐	☐	☐	☐	☐
Oxygen Biotherapeutics (Formerly SBI)	☐	☐	☐	☐	☐	☐	☐	☐	☐	☐	☐	☐	☐	☐
Pfizer	☐	☐	☐	☐	☐	☐	☐	☐	☐	☐	☐	☐	☐	☐
Popluation Health Research Institute	☐	☐	☐	☐	☐	☐	☐	☐	☐	☐	☐	☐	☐	☐
Portola Pharmaceutical	☐	☐	☐	☐	☐	☐	☐	☐	☐	☐	☐	☐	☐	☐
Procyrion Inc	☐	☐	☐	☐	☐	☐	☐	☐	☐	☐	☐	☐	☐	☐
Reflexion Health	☐	☐	☐	☐	☐	☐	☐	☐	☐	☐	☐	☐	☐	☐
Regeneron Pharmaceuticals, Inc.	☐	☐	☐	☐	☐	☐	☐	☐	☐	☐	☐	☐	☐	☐
Research Triangle Institute	☐	☐	☐	☐	☐	☐	☐	☐	☐	☐	☐	☐	☐	☐
ResMed	☐	☐	☐	☐	☐	☐	☐	☐	☐	☐	☐	☐	☐	☐
RespireRX Pharmaceuticals	☐	☐	☐	☐	☐	☐	☐	☐	☐	☐	☐	☐	☐	☐

Roche Diagnostics	☐	☐	☐	☐	☐	☐	☐	☐	☐	☐	☐	☐	☐	☐
Roche Diagnostics Corp	☐	☐	☐	☐	☐	☐	☐	☐	☐	☐	☐	☐	☐	☐
Roche Group	☒	☐	☐	☐	☐	☐	☐	☐	☐	☐	☐	☐	☐	☐
Sanofi-Aventis	☒	☐	☐	☐	☐	☐	☐	☐	☐	☐	☐	☐	☐	☐
Shionogi Inc	☐	☐	☐	☐	☐	☐	☐	☐	☐	☐	☐	☐	☐	☐
Society of Thoracic Surgeons	☐	☐	☐	☐	☐	☐	☐	☐	☐	☐	☐	☐	☐	☐
Stealth Peptides	☐	☐	☐	☐	☐	☐	☐	☐	☐	☐	☐	☐	☐	☐
Summit	☐	☐	☐	☐	☐	☐	☐	☐	☐	☐	☐	☐	☐	☐
Swedish Orphan Biovitrum AB	☐	☐	☐	☐	☐	☐	☐	☐	☐	☐	☐	☐	☐	☐
Tacere Therapeutics	☐	☐	☐	☐	☐	☐	☐	☐	☐	☐	☐	☐	☐	☐
Taiwanj Pharmaceuticals Company Ltg	☐	☐	☐	☐	☐	☐	☐	☐	☐	☐	☐	☐	☐	☐
The Medicines Company	☐	☐	☐	☐	☐	☐	☐	☐	☐	☐	☐	☐	☐	☐
Theravance Biopharma	☐	☐	☐	☐	☐	☐	☐	☐	☐	☐	☐	☐	☐	☐
Thrombosis Research Institute	☐	☐	☐	☐	☐	☐	☐	☐	☐	☐	☐	☐	☐	☐
Tokalas Inc	☐	☐	☐	☐	☐	☐	☐	☐	☐	☐	☐	☐	☐	☐
University of Michigan	☐	☐	☐	☐	☐	☐	☐	☐	☐	☐	☐	☐	☐	☐
Verily Life Sciences	☐	☐	☐	☐	☐	☐	☐	☐	☐	☐	☐	☐	☐	☐
Zimmer Biomet Holdings Inc	☐	☐	☐	☐	☐	☐	☐	☐	☐	☐	☐	☐	☐	☐
St. Jude Medical	☒	☐	☐	☐	☐	☐	☐	☐	☐	☐	☐	☐	☐	☐
	☐	☐	☐	☐	☐	☐	☐	☐	☐	☐	☐	☐	☐	☐
	☐	☐	☐	☐	☐	☐	☐	☐	☐	☐	☐	☐	☐	☐
	☐	☐	☐	☐	☐	☐	☐	☐	☐	☐	☐	☐	☐	☐
	☐	☐	☐	☐	☐	☐	☐	☐	☐	☐	☐	☐	☐	☐

☐ No Conflicts to report

Column Explanations:

(1) Funding is provided for my effort in research grants or contracts for multi-center projects in which I serve as either the Chairman, Co-Chairman, Executive or Steering Committee member or I am involved as a DCRI faculty leader for specific aspects in a project. A check in this column denotes that I am involved in such a research contract. Payments for the work performed for these contracts are made to the University. A portion of my salary as a faculty member, representative of % effort on the project, is supported by these funds.

(2) A research grant from this company supports the expenses associated with projects in the DUHS that are under my direction.

(3) I provide educational activities or deliver occasional lectures that generate revenue from this company. All revenues from these activities are paid to the University. None of it is taken for personal income. The three columns represent annual payments of more than $5,000, between $5,000 and $25,000, and in excess of $25,000 per year from this company.

(4) I receive personal income outside of my University salary for consulting or other services (Including CME) from this company. The three columns represent annual payments of more than $5,000, between $5,000 and $25,000, and in excess of $25,000 per year from this company.

(5) I receive personal income outside of my University salary for consulting or other non-CME services from this company. The three columns represent annual payments of more than $5,000, between $5,000 and $25,000, and in excess of $25,000 per year from this company.

(6) I personally receive royalties in excess of $5,000 per year from this company.

(7) I, or an immediate member of my family, have equity (with shareholder voting privileges) in this company of either > $5,000 or > 1%.
